# Creatine kinase-(MB) and hepcidin as candidate biomarkers for early diagnosis of pulmonary tuberculosis: a proof-of-concept study in Lambaréné, Gabon

**DOI:** 10.1007/s15010-022-01760-8

**Published:** 2022-02-08

**Authors:** Paulin N. Essone, Bayode R. Adegbite, Marien J. M. Mbadinga, Armel V. Mbouna, Fabrice Lotola-Mougeni, Ayodele Alabi, Jean R. Edoa, Bertrand Lell, Abraham S. Alabi, Ayola A. Adegnika, Michael Ramharter, Joel F. D. Siawaya, Martin P. Grobusch, Peter G. Kremsner, Selidji T. Agnandji

**Affiliations:** 1 Centre de Recherches Médicales de Lambaréné, Lambaréné, Gabon; 2 Institut für Tropenmedizin, Universität Tübingen and German Center for Infection Research Tübingen, Tübingen, Germany; 3 Unité de Recherche et de Diagnostics Spécialisés, Laboratoire National de Santé Publique/Centre Hospitalier Universitaire Mère Enfant Fondation Jeanne EBORI, Libreville, Gabon; 4 Center of Tropical Medicine and Travel Medicine, Department of Infectious Diseases, Amsterdam University Medical Centers, location Amsterdam, Amsterdam Infection & Immunity, Amsterdam Public Health, University of Amsterdam, Amsterdam, The Netherlands; 5 Department of Parasitology, Leiden University Medical Center, Leiden, The Netherlands; 6 Division of Infectious Diseases and Tropical Medicine, Department of Medicine 1, Medical University of Vienna, Vienna, Austria; 7 Department of Tropical Medicine, Bernhard Nocht Institute for Tropical Medicine and Department of Internal Medicine I, University Medical Center Hamburg-Eppendorf, Hamburg, Germany; 8 The African Society of Clinical Trials and the African Congress for Clinical Trials, Lambaréné, Gabon

**Keywords:** Biomarkers, Tuberculosis, Diagnosis, Creatine kinase-MB, Hepcidin

## Abstract

**Background:**

The present study aimed to evaluate the diagnostic utility of creatine kinase-MB (CK-MB), hepcidin (HEPC), phospholipase A2 group IIA (PLa2G2A), and myosin-binding protein C (MYBPC1) for tuberculosis (TB). These four biomarkers are differentially regulated between quiescent Mycobacterium tuberculosis (Mtb) infected individuals (non-progressors to TB disease) and Mtb-infected TB disease progressors 6 months before the onset of symptoms.

**Methods:**

We enrolled samples from patients experiencing moderate-to-severe pulmonary infections diseases including 23 TB cases confirmed by smear microscopy and culture, and 34 TB-negative cases. For each participant, the serum levels of the four biomarkers were measured using ELISA.

**Results:**

The levels of CK-MB and HEPC were significantly reduced in patients with active TB disease. CK-MB median level was 2045 pg/ml (1455–4000 pg/ml) in active TB cases and 3245 pg/ml (1645–4000 pg/ml) in non-TB pulmonary diseases. Using the receiver operating characteristic curve (ROC) analysis, HEPC and CK-MB had the Area Under the Curve (AUC) of 79% (95% CI 67–91%) and 81% (95% CI 69–93%), respectively. Both markers correlated with TB diagnosis as a single marker. PLa2G2A and MYBPC1 with AUCs of 48% (95% CI 36–65%) and 62% (95% CI 48–76%) did not performed well as single biomarkers. The three markers’model (CK-MB-HEPC-PLa2G2A) had the highest diagnostic accuracy at 82% (95% CI 56–82%) after cross-validation.

**Conclusion:**

CK-MB and HEPC levels were statistically different between confirmed TB cases and non-TB cases. This study yields promising results for the rapid diagnosis of TB disease using a single marker or three biomarkers model.

**Supplementary Information:**

The online version contains supplementary material available at 10.1007/s15010-022-01760-8.

## Introduction

About 10 million individuals develop tuberculosis (TB) every year, causing 1.3 million deaths per year [1]. The limited performance and reduced accessibility of diagnostic methods contribute to the high morbidity and mortality of TB [2]. Immunodiagnostic techniques such as point-of-care tests could substantially improve TB control. The tuberculin skin test (TST) and interferon (IFN)-g release assays (IGRA) cannot distinguish between TB disease and other respiratory infections [3]. Cytokine secretion profiles derived from antigen stimulation have been evaluated as immune diagnostic tests for TB. Various Mycobacterium tuberculosis (Mtb) antigens [4] and cytokine/chemokines other than IFN-g have been studied [5, 6]. Overnight incubation and other technical hurdles requested by the Mtb antigen-stimulated cytokine biosignatures test may be challenging in resource-limited settings. The identification of host markers from serum samples that have a more specific action in the host responses for the development of a rapid point-of-care test for TB disease remains, as of today, a by-and-large unmet medical need.

In a recent prospective cohort study of about 6000 quiescent Mtb-infected individuals, the prognostic utility of more than 3000 plasma proteins was evaluated [7]. A total of 42 individuals developed TB disease within 2 years (TB progressors) in this study. Proteins were quantified in plasma with a highly multiplexed proteomic assay (SOMAscan); 361 proteins including creatine kinase myocardial band (CK-MB), hepcidin (HEPC), phospholipase A2 group IIA (PLa2G2A) and myosin-binding protein C (MYBPC1) were differentially expressed between TB progressors and non-progressors before the onset of symptoms.

Hepcidin is a regulator of iron metabolism, which controls the expression of the transmembrane protein ferroportin. This transmembrane protein reduces the plasma level of iron by increasing iron intake by macrophages and limiting iron absorption during food intake [8]. A high plasma level of hepcidin will increase ferroportin expression and induces high concentration of iron within macrophages. A reduced hepcidin level will lead to low iron quantity inside macrophages and prevents Mtb growth [9–11]. An elevated hepcidin level is associated with disseminated TB disease, anaemia, and poor prognosis in patients with HIV-associated tuberculosis [12]. CK-MB is routinely used as a biomarker of myocardial infarction. An elevated level of this biomarker is an indicator of cardiac muscle damage [13]. The level of cardiac muscle damage during TB disease is unknown, although myopericarditis is common in TB patients [14]. PLa2G2A is an extracellular enzyme catalyzing the hydrolysis of the sn-2 fatty acid acyl ester bond of phosphoglycerides[15]. It is thought to participate in the regulation of phospholipid metabolism in biomembranes. A high concentration of this protein has been associated with coronary heart disease, rheumatoid arthritis and asthma. [16, 17]. MYBPC1 is a smooth skeletal muscle-specific enzyme. It plays an essential role in muscle contraction by recruiting muscle-type creatine kinase to myosin filaments[18]. This protein has been associated with breast cancer prognosis[19]. The functions of PLa2G2A and MYBPC1 during TB have not been studied. The present study aims to evaluate the secretion levels of CK-MB, HEPC, PLa2G2A and MYBPC1 in active TB disease and other pulmonary disease patients to determine their diagnostic utility.

## Materials and methods

### Study design

We conducted a cross-sectional study, enrolling samples received at the Centre de Recherches Médicales de Lambaréné from January 2019 to December 2020 for TB testing. Samples from participants experiencing TB-suggestive symptoms and providing written informed consent were enrolled to evaluate the diagnostic utility of selected biomarkers in resource-constrained settings using the method developed in previous studies [20–22].

### Study participants and procedure

Adults (≥ 18 years) with TB-suggestive symptoms who attended the TB laboratory of CERMEL or were hospitalised in one of the Lambaréné hospitals were consecutively screened. Patients who were unable to provide informed consent were excluded. For volunteers, written informed consent was obtained, and a structured questionnaire addressing sociodemographic and clinical information was administered. All patients provided two sputum samples (one during the outpatient/inpatient attendance and one in the early morning the day after) as suggested by the national tuberculosis control programme and described elsewhere [23]. Participants with positive smear microscopy and positive Mtb culture results were defined as TB cases, while non-TB patients were defined as smear- and culture-negative. Whole blood collected in a dried tube for each participant was centrifuged, and serum was collected into several aliquots and stored at − 80 °C. The individual biomarker secretion levels were compared between confirmed TB cases and non-TB patients.

### Biomarkers selection process

The four biomarkers of interest in our study were selected from a list of proteins reported by Penn-Nicholson et al. [7] as potential predictors of TB disease in participants with quiescent Mtb. More than 6000 Mtb infected HIV-negative participants were followed for 2 years and samples actively and passively collected from onset of TB disease. Participants were categorized as TB progressors if they developed TB during the follow-up period, and as TBnon-progressors if they did not develop TB disease. The expression profiles of more than 3000 proteins were compared between TB disease progressors and non-progressors using SomaLogic technology [7]. This platform is based on modified-aptamer binding technology (SOMAmers) that bind to their respective target proteins to determine their presence and quantity in a sample. The performances of each protein, including p value, fold change, and False Discovery Rate (FDR), are provided in Supplementary Table 1. Applying strict selection criteria, we selected proteins with a significant p value, a fold change difference of a least 1.4 and a False Discovery Rate (FDR) of less than 0.05 as potential TB diagnostic candidates. A reduced list of 31 proteins differentially expressed between TB progressors and non-progressors was obtained (Supplementary Table 1). We selected four biomarkers (CK-MB, HEPC, PLa2G2A and MYBPC1) for evaluation in this pilot study due to their potential role in expressing anti-TB properties of macrophages (the most abundant cell population in tuberculous granuloma) [24].

### ELISA

The serum levels of CK-MB, HEPC, PLa2G2A and MYBPC1 were measured using ELISA kits (MyBioSource, San Diego, USA). ELISAs were performed according to the manufacturer's instructions. Diluted standards, blanks and 5X diluted samples were added into the appropriate pre-coated plates. The plates were incubated for 90 min at 37 °C. A hundred (100) µl of detection solution A was added to each well plate and set for 45 min. The plates were washed three times before adding 100 µl of detection solution B and incubation for an additional 45 min. The plates were then washed five times, and 90 µl of substrate solution was added and incubated in the dark for 15 min. 50 µl of stop solution was added to each well and read at 45 nm using the ELISA reader.

### Statistical analysis

Comparison of the two study groups (TB vs no TB) was performed using the Chi-square (Chi2) for women and men proportions,and Kruskall-Wallis test for the distribution of age and BMI. The accuracy of all biomarkers for the diagnosis of TB disease was estimated by performing receiver operator characteristics (ROC) curve analysis. The utility of combinations of biomarkers for TB diagnostic was investigated by performing best subsets general discriminant analysis (GDA), with leave-one-out cross-validation (where all data were individually validated). Data were analysed using the R software, version 4.0.3 and GraphPad prism, version 5.0 (GraphPad Software, San Diego, CA, USA).

## Results

### Study participants

Fifty-seven (57) participants were enrolled in this study, including 23 confirmed TB cases and 34 non-TB cases (Table 1). The median age of included participants was 44 [IQR 22–59]. Weight, sex and height were evenly distributed between the two study groups.

**Table 1:** Demographic characteristics of study participants

	All	No TB	TB	*P* value
	*N* = 62	*N* = 36	*N* = 26	
Gender				0.900
Women (*N*, %)	28 (45.2)	17 (47.2)	11 (42.3)	
Men (*N*, %)	34 (54.8)	19 (52.8)	15 (57.7)	
Age median [IQR]	43.9 [22.2–59.2]	49.2 [23.2–62.6]	34.1 [22.3–49.6]	0.077
BMI, median [IQR]	20.1 [16.7–22.1]	20.9 [17.2–24.3]	19.7 [16.5–20.9]	0.180

Chi^2^ test was used to compare women and men proportion, and Kruskall-Wallis test was used to compare the distribution of the Age and the BMI acconding to TB status

*BMI* body mass index, *IQR* interquartile range

### The utility of single biomarkers in discriminating TB from alternative pulmonary diseases

**Fig. 1:**
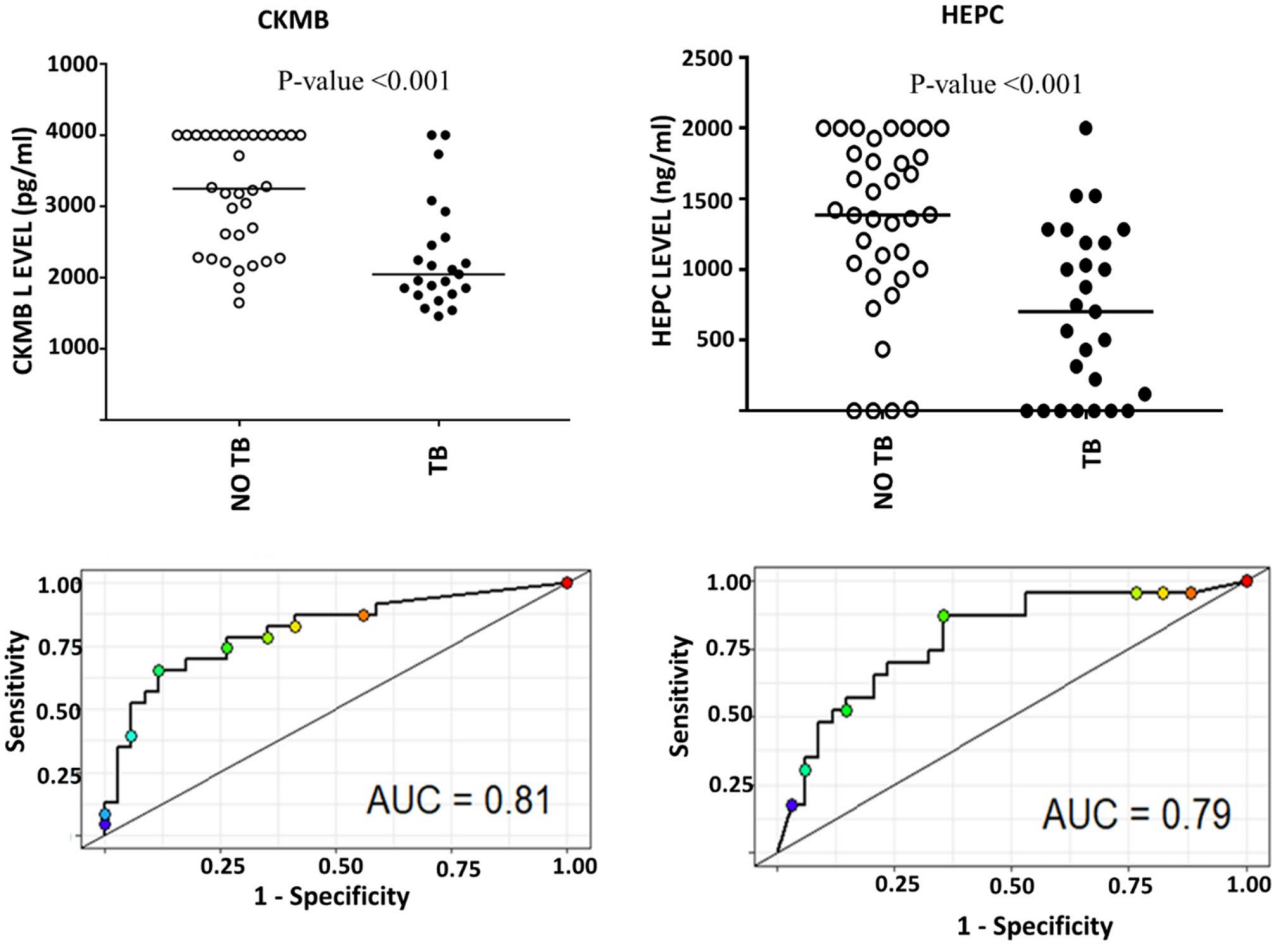
Scatter-dot plots and ROC curves of analytes discriminating TB disease to alternative pulmonary diseases. Representative dot plots showing the levels of analytes in serum and ROC curves for accuracy in the diagnosis of TB disease. Error bars in the dot plots represent the median analyte levels

The serum levels of MYBPC1, PLa2G2A, CK-MB and HEPC were compared between confirmed TB cases and pulmonary infections other than TB. All four biomarker candidates were detected in both study groups (Fig. 1). Serum levels of CK-MB and HEPC were significantly reduced in TB cases (*P* < 0.001) compared to non-TB. The diagnostic accuracy of the four biomarkers was evaluated by ROC curve analysis. The area under the ROC curve (AUC) of MYBPC1 and PLa2G2A were 0.48 and 0.62, respectively, indicating a low diagnostic accuracy. PLa2G2A has the poorest diagnostic utility with sensitivity and specificity around 50% (cut off = 1.19 ng/ml). With an AUC of 0.62 (62%), MYBPC1 has a sensitivity of 70% but a reduced specificity of 61% for a cut-off at 26.98 ng/ml. CK-MB and HEPC yielded AUCs of 81% and 79%, respectively (Fig. 1, Table 2). For a selected cut-off at 515.9 ng/ml (optimal sensitivity and specificity), CK-MB had a sensitivity of 78.3% and specificity of 73.5%, respectively. The sensitivity and specificity of HEPC were 87% and 64%, respectively, for a cut-off at 130.6 ng/ml.

**Table 2:** Diagnostic potential of single host markers

Host markers	TB (range)	No TB (range)	*P*-value	AUC % (95% CI)	Sensitivity (95% CI)	Specificity (95% CI)	Cut off value
MYBPC1 (ng/ml)	170 (70–500)	125 (70–500)	0.3	62 (48–76)	70 (50–86)	61 (43–77)	> 26.98
PLa2G2A (pg/ml)	0 (0–2000)	0 (0–2000)	0.8	48 (36–65)	48 (29–68)	56 (38–72)	> 1.19
CK-MB (pg/ml)	2045 (1455–4000)	3245 (1645–4000)	**< 0.001**	**81** (69–93)	78.3 (56–93)	73.5 (56–87)	< 515.9
HEPC (ng/ml)	375 (0–1000)	700 (0–1000)	**< 0.001**	**79** (67–91)	87 (66–97)	64 (46–80)	< 130.6

Median levels (and ranges in parenthesis) of serum analytes and their accuracy in the diagnosis of TB disease as a single host marker. *P* values were calculated using the Mann Whitney *U* test

*AUC* area under the receiver operator characteristics curve, *95% CI* 95% confidence interval

### The utility of 2- to 3-biomarker models in the rapid diagnosis of TB disease

**Fig. 2:**
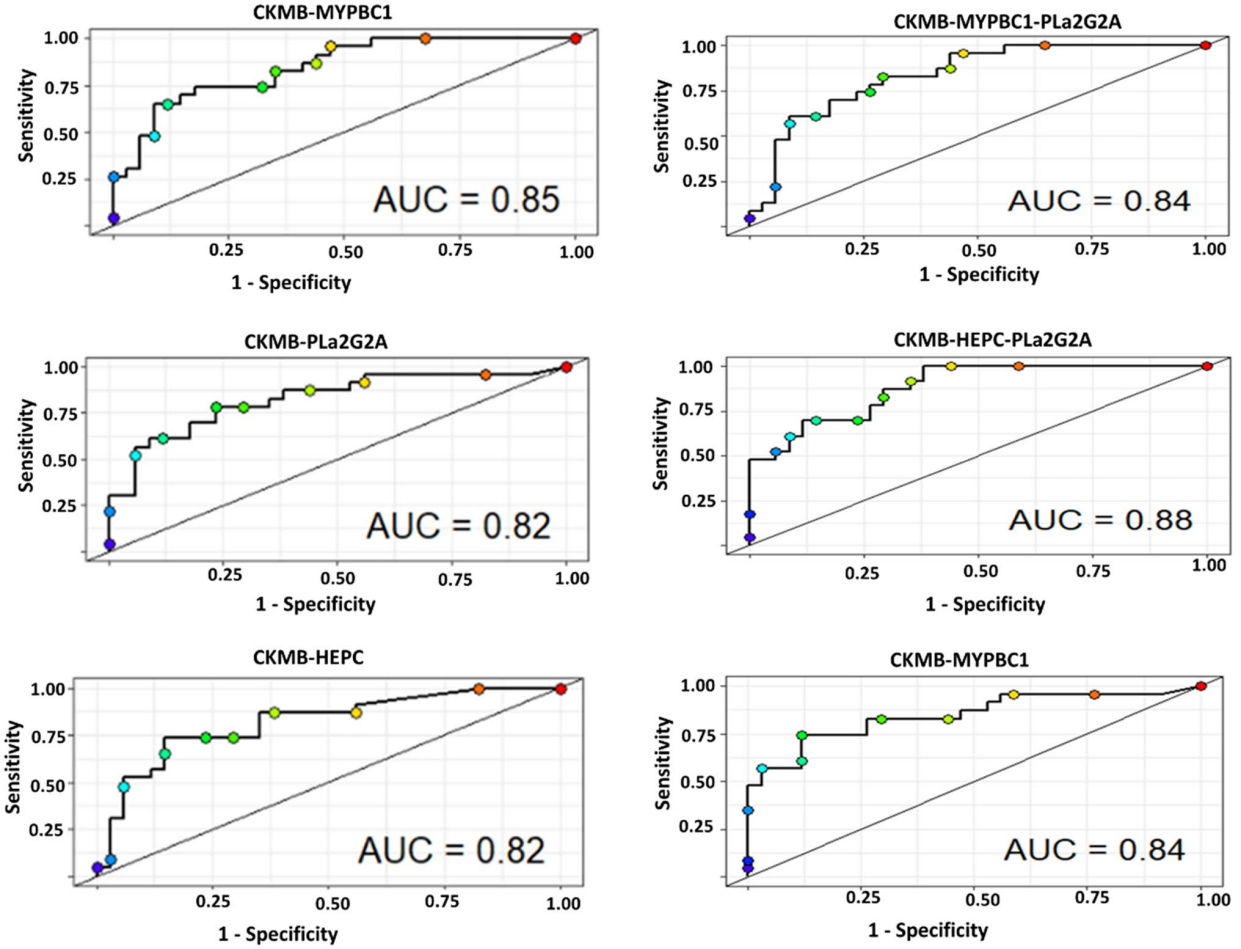
ROC curves analysis of the most discriminative two and tree markers models

The diagnostic accuracy of TB disease was evaluated using several models. CK-MB and HEPC had respective AUCs of 81% and 79% as single markers. When the two most accurate single biomarkers CK-MB and HEPC were tested as a model, their diagnostic accuracy increased to 82%. After leave-one-out cross-validation analysis, the accuracy of this model was found to be 75.4%. The CK-MB-HEPC model successfully classified (after leave-one-out cross-validation) 76.5% non-TB and 73.9% TB cases, respectively (Fig. 2 and Table 3). The diagnostic accuracy of MYBPC1 was low as a single biomarker. This biomarker was associated with CK-MB in a 2-biomarkers model, and it had the highest diagnostic accuracy (AUC of 85%) of all 2-biomarkers models. However, the diagnostic accuracy of this model was significantly reduced to 70.2% after leave-one-out cross-validation analysis.

**Table 3:** Utility of combination of analytes in the diagnosis of TB disease

Combinations	Leave-one-out cross validation			AUC	Wilks	*f*	*P*-value
	TB cases (%)	No TB (%)	Accuracy (%)				
CKMB, HEPC	73.9	76.5	75.4 [62.2–85.9]	82	0.29	65.1	< 0.001
MYBPC1, HEPC	47.8	82.4	68.4 [54.8–80.1]	79	0.47	30.4	< 0.001
MYBPC1, CKMB	73.9	67.6	70.2 [56.6–81.6]	85	0.28	70.9	< 0.001
PLa2G2A, CKMB, HEPC	73.9	88.2	82.5 [70.1–91.3]	84	0.34	33.8	< 0.001
MYBPC1, CKMB, HEPC	69.6	79.4	75.4 [62.2–85.9]	88	0.33	35.6	< 0.001
MYBPC1, PLa2G2A, HEPC	47.8	85.3	70.2 [56.6–81.6]	78	0.48	19.3	< 0.001
MYBPC1, PLa2G2A, CKMB	73.9	76.5	75.4 [62.2–85.9]	84	0.29	42.7	< 0.001

The AUC of 3-biomarker models varied between 78 and 88%. The CK-MB-HEPC-MYBPC1 model was the most accurate with an AUC of 88% but dropped to an AUC of 75.4% after leave-one-out cross-validation analysis.

The CK-MB-HEPC-PLa2G2A model had an AUC above 82% after leave-one-out cross-validation analysis. This 3-biomarker model successfully classified 88.2% of non-TB cases and 73.9% of active TB diseases.

## Discussion

Rapid diagnosis of active TB remains an unmet medical need due to the lack of good biomarkers. The current study shows that CK-MB and HEPC are differentially expressed in active TB cases compared to patients with pulmonary infections other than TB.

Previous studies have shown promising results with novel Mtb antigen-stimulated growth factors and cytokines, including epidermal growth factor (EGF), tumour necrosis factor-alpha (TNF-α), vascular endothelial growth factor (VEGF), interferon-gamma (IFN-γ, IL-12 and IL10 [5, 25]. These biomarkers were differentially expressed when comparing TB cases to healthy household contacts in a 7-day assay. In an overnight assay with alternative pulmonary diseases as controls (instead of healthy controls), none of these markers could differentiate TB from other pulmonary diseases [20, 26]. These studies have shown the limitation of cytokines/chemokines as biomarkers for the rapid diagnosis of TB disease.

The current study illustrates the utility of circulating CK-MB and HEPC levels for TB diagnosis. CK-MB is present in myocardial cells and is the most specific enzyme for clinical diagnosis of myocardial injury [27]. This enzyme has also been identified in patients presenting with a wide range of noncardiac diseases [28–30]. Although the link between CK-MB and infectious diseases is not fully understood, it has been detected in patients with infectious diseases, including dengue infection, in the absence of heart disease or myositis [31, 32]. The secretion level of CK-MB in pulmonary diseases has been investigated. A study performed in Japan investigating the level of CK-MB in different lung carcinomas has shown a low secretion level of this marker [33]. Several studies showed increased secretion of CK-MB in patients with Severe Acute Respiratory Syndrome Coronavirus-2 (SARS‐CoV‐2) [34]. The secretion level of this enzyme was associated with the severity and case fatality rate during SARS‐CoV‐2 infection [35, 36]. Increased levels of CK-MB in SARS‐CoV‐2 patients with mild infection has also been reported [37]. CK-MB is not specifically secreted during TB disease, but its expression profile could help the rapid diagnosis of active TB compared to alternative pulmonary infections.

Hepcidin (HEPC) is an antimicrobial-like peptide hormone; defined as the master regulator of iron metabolism. We found a reduced level of this hormone in TB patients compared to pulmonary infections other than TB patients in the current study. Mtb infection of mice resulted in decreased levels of hepcidin [38]. Hepcidin regulates iron through modulation of the iron transporter ferroportin highly expressed in macrophages. Hepcidin reduces the circulating level of iron by increasing iron intake into macrophages and limited dietary iron absorption [8, 39]. Iron is essential for bacteria growth. A high concentration of hepcidin during Mtb infection may lead to a high concentration of iron in infected macrophages and favour intracellular growth of the bacteria, suggesting a potential role of HEPC in regulating the anti-TB function of lung-resident macrophages. Hepcidin is stable in healthy individuals, and it is not influenced by age. An elevated level of hepcidin in HIV-infected individuals is a prognostic biomarker of TB in this population [12]. A high serum concentration of this biomarker has been positively correlated to a late time to Mtb culture conversion [9]. The interpretation of hepcidin levels during TB disease may depend on malaria infection in malaria-endemic areas. Data have shown that febrile malaria increases the serum level of hepcidin while severe and complicated malaria is associated with reduced plasma levels in African children [9]. Particular attention should be paid to the co-morbidity of TB-malaria when interpreting hepcidin results. The present study shows the potential utility of hepcidin and CK-MB as biomarkers for diagnosing TB disease. Although these biomarkers are secreted in most pulmonary conditions, they may have a particular secretion profile during TB. They could help in the development of TB rapid point-of-care tests.

Healthy participants and quiescent Mtb infected individuals were not enrolled in our study. Their presence may have added information rendering interpretation of our results possibly less straightforward. However, quiescent Mtb infection is not routinely tested in high-endemic countries like Gabon and has no impact on public health strategy. Despite these potential limitations, we think that the study design responded to the study's main objective to assess the diagnostic utility of the selected biomarker.

The selection of four biomarkers out of 31 identified in the predictive utility study may appear arbitrary, but was based on their high value as disease progression markers and on our assumption that they play roles in the anti-TB functions of the lung-resident macrophages. Further steps towards a large and multicenter validation trial require (1) a mechanistic confirmatory study of the roles of CK-MB and hepcidin in the regulation of anti TB functions of lung-resident macrophages. (2) a study that assesses the diagnostic utility, mode of action and standard ranges of an additional list of biomarkers.

## Conclusion

The present study demonstrates potential, warranting further exploration of CK-MB and hepcidin in the rapid diagnosis of TB disease. CK-MB and hepcidin were differentially expressed in active TB diseases when compared to pulmonary diseases other than TB, presenting a good AUC as a single marker. The current study is a proof-of-concept in which the results require validation in a more extensive and multicenter study population. The diagnostic performance of of these biomarkers in specific population like smear-negative TB and HIV-TB patients need to be investigated.

## Supplementary Information

Below is the link to the electronic supplementary material.

Supplementary file1 (XLSX 688 KB)

## Data Availability

All original data will be made available on request by the correcponding authors.
